# For Eczema of the Anus

**Published:** 1890-03

**Authors:** 


FOR ECZEMA OF THE ANUS.—Shoemaker of Philadelphia, recommends the following mixture for eczema of the anus: Take of fluid extract of witch-hazel, 2 ounces; glycerine, 2 ounces. Mix. Dose, from one-half to one teaspoonful night and morning. Rectal injections of the same combination can be made with advantage once or twice a day.—*Medical Index.*

